# ΔN-P63α and TA-P63α exhibit intrinsic differences in transactivation specificities that depend on distinct features of DNA target sites

**DOI:** 10.18632/oncotarget.1845

**Published:** 2014-03-23

**Authors:** Paola Monti, Yari Ciribilli, Alessandra Bisio, Giorgia Foggetti, Ivan Raimondi, Paola Campomenosi, Paola Menichini, Gilberto Fronza, Alberto Inga

**Affiliations:** ^1^ Mutagenesis Unit, Istituto di Ricerca e Cura a Carattere Scientifico Azienda Ospedaliera Universitaria San Martino-IST-Istituto Nazionale per la Ricerca sul Cancro, Genoa, Italy;; ^2^ Laboratory of Transcriptional Networks, Centre for Integrative Biology, CIBIO, University of Trento, Trento, Italy;; ^3^ Department of Biotechnology and Life Sciences, DBSV, University of Insubria, Varese, Italy;; ^4^ The Protein Factory, Centro Interuniversitario di Ricerca in Biotecnologie Proteiche, Politecnico di Milano, ICRM-CNR Milano and Università degli Studi dell'Insubria, Varese, Italy

**Keywords:** *TP63*, transcription, transactivation specificity, *TP73*, *TP53*, Response Element

## Abstract

*TP63* is a member of the *TP53* gene family that encodes for up to ten different TA and ΔN isoforms through alternative promoter usage and alternative splicing. Besides being a master regulator of gene expression for squamous epithelial proliferation, differentiation and maintenance, P63, through differential expression of its isoforms, plays important roles in tumorigenesis. All P63 isoforms share an immunoglobulin-like folded DNA binding domain responsible for binding to sequence-specific response elements (REs), whose overall consensus sequence is similar to that of the canonical p53 RE. Using a defined assay in yeast, where P63 isoforms and RE sequences are the only variables, and gene expression assays in human cell lines, we demonstrated that human TA- and ΔN-P63α proteins exhibited differences in transactivation specificity not observed with the corresponding P73 or P53 protein isoforms. These differences 1) were dependent on specific features of the RE sequence, 2) could be related to intrinsic differences in their oligomeric state and cooperative DNA binding, and 3) appeared to be conserved in evolution. Since genotoxic stress can change relative ratio of TA- and ΔN-P63α protein levels, the different transactivation specificity of each P63 isoform could potentially influence cellular responses to specific stresses.

## INTRODUCTION

*TP63* is a member of the *TP53* gene family [1] that encodes for up to ten different TA- and ΔN- isoforms (α, β, γ, δ, ε) through differential promoter usage and alternative splicing [2]. The TA isoforms contain the N-terminal transactivation domain (TA1), whereas the ΔN isoforms are transcribed from an internal promoter (P2) and lack the entire TA1 domain. A second C-terminal transactivation domain (TA2), present in all P63α and β isoforms, has been reported [3].

P63 is a master regulator of gene expression for squamous epithelial proliferation, differentiation and maintenance; in fact, heterozygous germline *TP63* mutations are causative for a subset of human ectodermal dysplasia syndromes, confirming the key role of P63 in epidermis and limb formation during development [4]. The ΔN-P63α isoform is the variant predominantly expressed in basal epithelial cells of skin, breast, prostate and urinary tract, its inactivation being associated with the developmental defects. Conversely, TA-P63 isoforms are barely detectable in the same tissues and have instead been identified in the female germ line [5-7], where they play a role in quality control in response to DNA damage through the modulation of apoptosis [8].

Since the ΔN-P63 isoforms lack the N-terminal transactivation domain, it was originally proposed that these proteins might act primarily as oncogenes through dominant-negative mechanisms [1]. Indeed, these isoforms can play an important role in the progression of triple negative breast cancers where ΔN-P63α promotes tumor survival by inhibiting TA-P73 pro-apoptotic activity [9]. However, different studies indicate that ΔN-P63 can be transcriptionally active [3, 10-13]. For example, ΔN-P63α may directly contribute to tumorigenesis by up-regulating the expression of the chaperone protein HSP70, which displays proliferative and anti-apoptotic functions [14] or by repressing pro-apoptotic genes [15]. However, ΔN-P63 isoforms may have also tumor suppression function; in fact ΔN-P63α has been shown to transcriptionally activate genes like *VDR* and *ID-3*, causing a decrease in cell invasion [16, 17].

The TA-P63 isoforms have been described to induce cell cycle arrest, senescence and apoptosis [18, 19]. Recent evidence suggested that TA-P63 also inhibits metastasis either by transcriptionally activating metastasis suppressor genes, such as *BHLHE41* (Sharp1) and *CCNG2* (Cyclin G2) [20] or by directly up-regulating miR-130b and the microRNA processing enzyme Dicer [21].

Regulation of target genes expression by P63 is achieved through the binding to sequence-specific REs whose consensus sequence is highly similar to that of p53 REs (RRRCWWGYYY-n-RRRCWWGYYY; R=purine; W=A/T; Y=pyrimidine; n=0-13 bp spacer; CWWG = CORE) [22]. Structures of P53, P63 and P73 DNA binding domains (DBDs) co-crystallized with DNA target sequences revealed overall conserved conformation and DNA-protein contact sites, even though subtle structural differences were observed [23-25]. *In vivo* DNA binding studies have demonstrated specific features in P53, P73 and P63 target sites' recognition [26-31], supporting the view that promoter selectivity contributes to the functional divergence that is apparent by genetic studies in mice or by germ line mutations in humans diseases.

In particular, relative to the P53 consensus, the P63 consensus RE was enriched in A/G at position 5 and C/T at position 16 [27]. Recently, the observation that REs containing two half-sites with one overlapping base pair were recognized by P63, but not by P53, provided additional evidence that specific recognition of *cis-*elements contribute to functional divergence among P53 family proteins [32]. In particular, ChIP-sequencing data from different cellular systems indicated a high correlation among P63-bound sites, the majority of which are not bound by P53 proteins activated by DNA damage response [33].

To examine the contribution of RE sequence features to P63-dependent transactivation specificity we took advantage of a functional assay in yeast where a single P63 isoform can be expressed and its capacity to stimulate transcription from isogenic promoter-reporter constructs measured [28, 34-36]. Being the promoter landscape constant except for the RE being tested, we anticipate that differences in transactivation potential could be directly related to the nature of the interactions of P63 protein with DNA target sites. From the results obtained with P63 proteins on more than 80 different REs, those obtained with P73 and P53 proteins for a selected group of REs, and also from the correlation between yeast- and mammalian-transcription assays, we uncovered that ΔN- and TA-P63α exhibit different transactivation specificities. These changes are dependent on specific features of the target RE sequences, were not observed with corresponding P73 and P53 proteins, and are likely related to intrinsic differences in the oligomeric state and in the cooperative DNA binding between ΔN- and TA-P63α proteins.

## RESULTS

### ΔN- and TA-P63α isoforms exhibit different transactivation specificities in a genetically defined yeast-based assay

**Figure 1:**
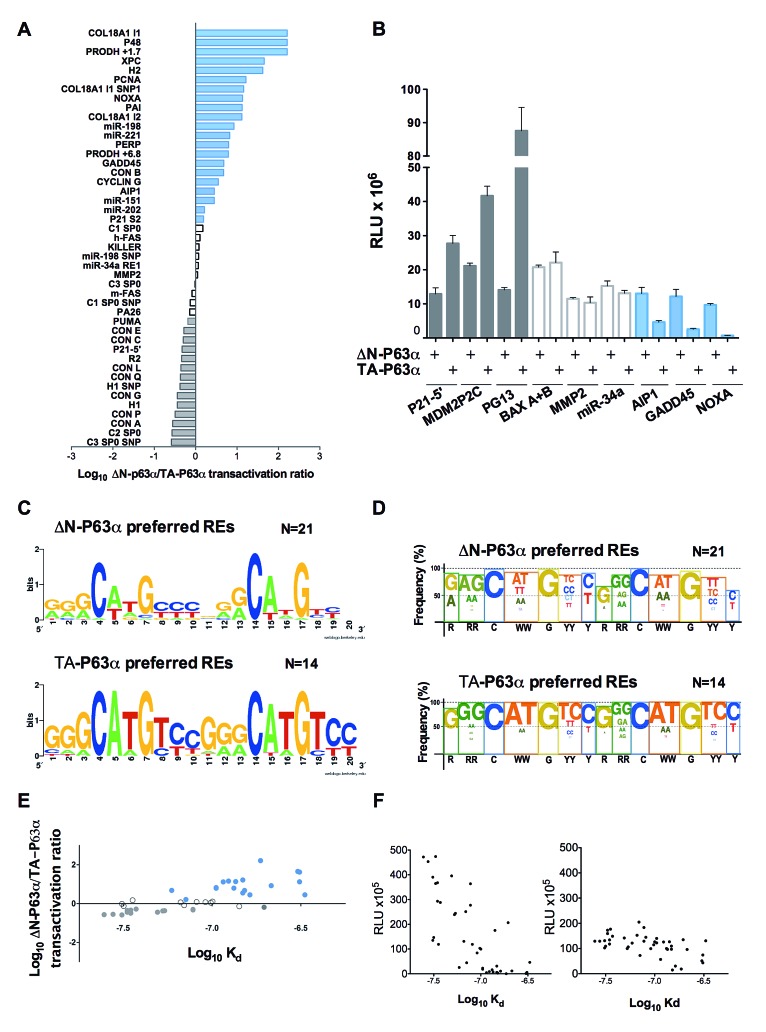
ΔN-P63α and TA-P63α exhibit differences in transactivation capacity and specificity A) Ratio between ΔN-P63α and TA-P63α transactivation towards 45 canonical REs. The names of the REs are listed on the left, while the sequences and feature information can be found in Table S1A. Results are plotted in a logarithmic scale. Grey or blue bars mark REs that were at least 1.5 times more responsive to TA-P63a or to ΔN-P63α, respectively (*i.e.* ΔN-P63α/TA-P63α ratio being lower than -0.17 or higher than 0.17 in log scale). Differences in ratio comprised in that interval were not considered as significant (white bars). The very high ratio for the top three REs is caused by extremely weak responsiveness to TA-P63α (see Table S1A). B) Example of the yeast-based transactivation assay. ΔN-P63α or TA-P63α were expressed under the *GAL1* inducible promoter (8 hours at 0.128% galactose) in isogenic yeast reporter strains containing the indicated P53-family REs. Light Units were normalized to the optical density of the cultures and also to the activity measured in strains transformed with an empty expression vector (RLU). Average and standard errors of four biological replicates are plotted. C) Traditional Logo view showing sequence features for the group of 21 REs that were more responsive to ΔN-P63α than to TA-P63α (see panel A and Table S1A) or for the 14 REs that were more responsive to TA-P63α than to ΔN-P63α (see panel A and Table S1A). The logo was generated with the WebLogo 3 tool (http://weblogo.berkeley.edu/logo.cgi). D) Custom logo summary highlighting dinucleotide motifs at the CWWG CORE and the RR or YY DNA contact sites. The height of the color box reflects the percentage of consensus bases at each position. Font size of consensus bases or motifs is proportional to their frequency of occurrence. The consensus sequence motif is indicated below the figure. E) Comparison of predicted DNA binding affinity for the p53 REs listed in Table S1A and the ratio of relative transactivation measured with TA-P63α and ΔN-P63α. Each dot represents a different RE sequence and the color matches the groupings based on the ΔN-P63α/ΤΑ-P63α transactivation ratio reported in panel A and in Table S1A, plotted on a logarithmic scale. F) Relative transactivation potential of ΔN-P63α (left panel) and ΤΑ-P63α (right panel) compared to the predicted values of Log K_d_ for all REs in panel E.

Initially, we compared TA-P63α and ΔN-P63α transactivation abilities towards 53 canonical P53 family REs using the yeast functional assay (Table S1A). The results clearly showed isoform-dependent transactivation capacity with the identification of groups of REs exhibiting different ΔN-P63α/TA-P63α activity ratios. Next to a group of 14 REs towards which TA-P63α was at least 1.5 times more active than ΔN-P63α (Figure 1A, gray bars; Table S1A, gray highlight), there was a group of 10 REs exhibiting quite similar responsiveness to the two isoforms (Figure 1A, white bars; Table S1A, white cells), followed by another group of 21 REs that was more responsive to ΔN-P63α (Figure 1A, blue bars; Table S1A, blue highlight). Eight REs were not active (Table S1A, green highlight). We then extended the functional analysis of P63α isoforms to 34 additional REs (canonical but containing a spacer sequence between the two decameric half-sites, or non-canonical, comprising three-quarter sites and half-sites, multimers or altered-structure REs) (Table S1B). The results confirmed the existence of groups of REs with different ΔN-P63α/TA-P63α activity ratios. As an example, results obtained with three selected REs from each of the different groups are presented in Figure 1B. The sequence features of the two groups of canonical REs (without spacer) (Table S1A) showing different ΔN-P63α/TA-P63α activity ratios were summarized using two logo views, the second of which takes into account the sequences of dinucleotide motifs flanking the conserved C and G bases of the RE consensus (Figures 1C and 1D). Overall, ΔN-P63α showed higher activity than TA-P63α towards REs containing a higher frequency of non-consensus bases and a reduced frequency of the CATG sequence at the CWWG core motif. The estimation of relative binding affinities (Log K_d_) for the canonical REs (Table S1A) based on a binding predictor matrix [37], in relation to the ΔN-P63α / TA-P63α activity ratio (Figure 1E) or to the transactivation capacity of each protein (Figure 1F), strongly supports the finding that TA-P63α exhibited higher transactivation potential but limited to higher affinity REs (Figure 1F, right panel). Conversely, ΔN-P63α exhibited a generally lower transactivation potential but towards a wider range of REs, including those with predicted lower K_d_ (Figure 1F, left panel). Moreover, the comparison of the relative transactivation capabilities of TA-P63α and ΔN-P63α on CON A (2xGGGCATGTCC) and CON B (2xGGGCTAGTCC) supports the impact of CWWG sequences in determining isoform specificity; in fact, the ratio of ΔN-P63α/TA-P63α responsiveness changed from 0.28 for CON A to 4.74 for CON B (Table S1A). Interestingly, differences in torsional flexibility were described for P53 consensus REs containing CATG or CTAG core motifs, and shown to impact on P53 binding cooperativity [38].

Regarding P63α isoforms activity on non-canonical sites, both ΔN-P63α and TA-P63α were inactive with half-site REs (Table S1B). Moreover, ΔN-P63α was slightly less sensitive than TA-P63α to the negative impact of spacers between half-sites and was more active towards three-quarter sites.

Overall, TA-P63α showed a transactivation preference towards high affinity REs while ΔN-P63α revealed a preference for REs predicted to have lower affinity, but high cooperativity [38].

### ΔN- and TA-P63β and P73 isoforms, as wll as P53 N-ter and C-ter variants do not exhibit different transactivation specificities in yeast

Given the unexpected differences in transactivation specificity observed with P63α variants at the N-terminal region, we extended the analysis to other P53 family proteins. Firstly, we measured TA-P63β and ΔN-P63β transactivation specificities using a selected subset of 35 REs (Table S2).

**Figure 2:**
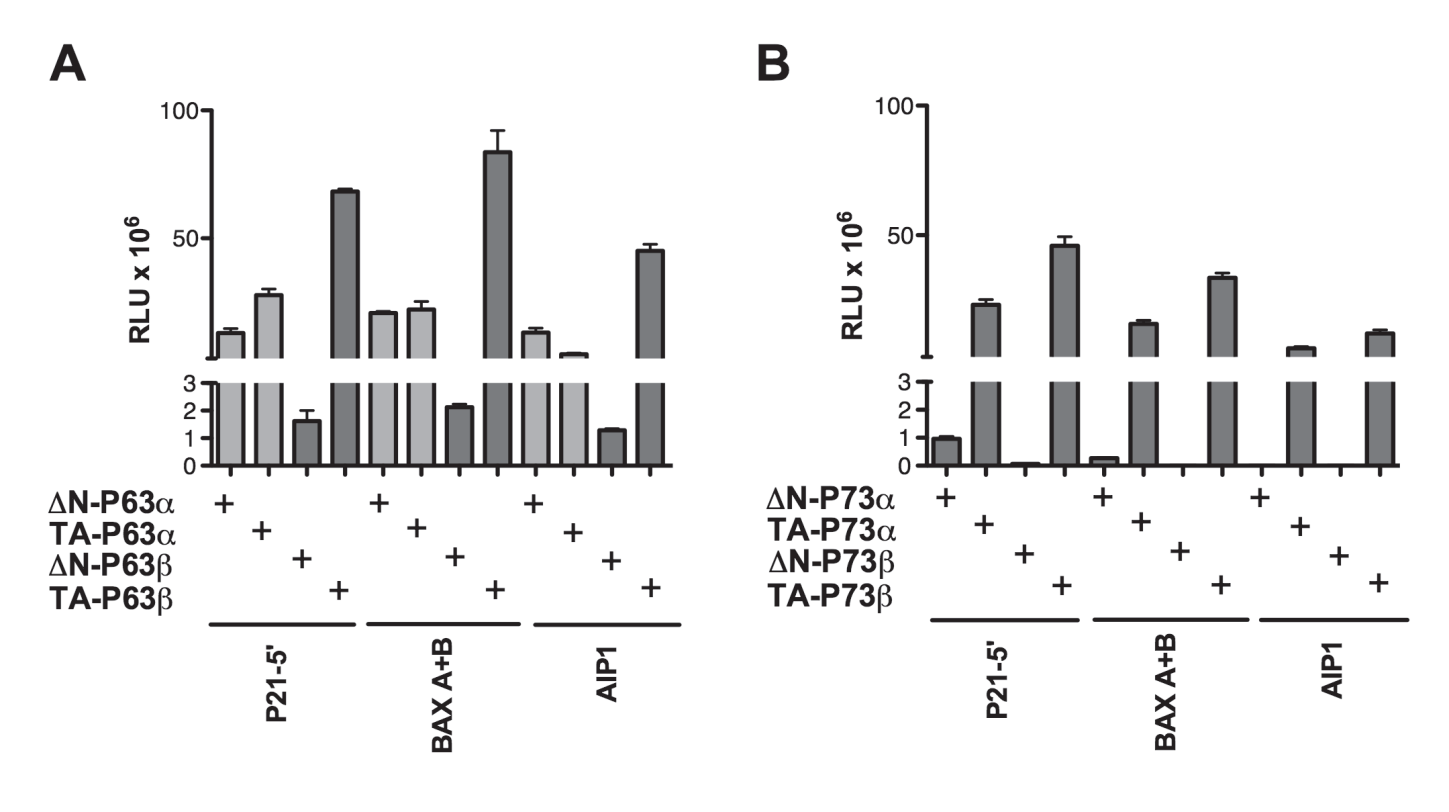
ΔN- and TA isoforms of P63β as well as P73α or P73β isoforms do not exhibit transactivation specificity in yeast The indicated P63 (A) or P73 (B) proteins were expressed from stable transformants of isogenic yeast reporter strains and relative transactivation was measured as described in Figure 1B. Average and standard errors of four biological replicates are plotted. In panel A, the light gray bars indicate results obtained with the P63 proteins that were presented in Figure 1 and are included here to facilitate the comparison of results obtained with the P63β isoforms. Additional results are presented in Table S2 and S3.

TA-P63β was more active than ΔN-P63β, with the exception of four REs (PRODH+4.7, PRODH-3.1, C1 SP2 and PRODH-0.9) (Table S2). TA-P63β also showed higher transactivation capacity than TA-P63α and appeared to be less sensitive to the deleterious impact of a spacer between half-sites, although it remained inactive on half-sites (Tables S1 and S2). On the contrary, ΔN-P63β exhibited weak activity on all tested REs, generally much lower than ΔN-P63α (Tables S1 and S2). Hence, there was no clear evidence for a change in specificity for TA- and ΔN-P63β unlike the case of the corresponding P63α isoforms (Figure 2A).

ΔN- and TA-P73α isoforms, along with the corresponding β isoforms, were also analyzed on a total of 14 selected REs (Table S3). As observed for P63 proteins, TA-P73β had a higher transactivation capacity than TA-P73α (Figure 2B). TA-P73α had always a much higher transactivation capacity than ΔN-P73α, the latter being barely active in yeast. Furthermore, ΔN-P73β was completely inactive in our experimental system.

In order to rule out that the observed heterogeneity in transactivation could be due to differences in protein steady-state levels, Western blot analysis was performed. Similar expression of TA-P63α and ΔN-P63α isoforms was observed in yeast (Figure S1), confirming previous results [39]. TA-P63β protein levels appeared to be lower than those of ΔN-P63β hence the higher activity of the TA-P63β is even underestimated. The inactive ΔN-P73β was expressed at fairly good levels.

To determine the relative transactivation potential of all the three P53 family gene members, the activity of P53 was also measured on a subset of 35 REs previously tested with P63 isoforms (Table S4). As expected, P53 was always a more potent transcription factor compared to P63 and P73 isoforms (TA, ΔN, α, β), and the only protein active with half-site REs in our assay. These data are in agreement with previous results and could be related, at least in part, to the higher relative expression of P53 proteins in yeast [28]. However, a group of five canonical REs (without spacer) was identified (P48, PERP, COL18A1, H2, miR-198) that exhibited comparable or even higher responsiveness to ΔN-P63α or TA-P63β than to P53 (Table S5). Considering REs where both ΔN-P63α/P53 and TA-P63β/P53 ratios were above the 1.5 threshold (P48, PERP and mir-198), we noticed an enrichment of CAAG sequences at core motif (3/5, 60%). In the case of the miR-198 RE, two single nucleotide changes from CAAG to CATG in both core motifs led to ~30 fold increase in responsiveness to P53 but only to a 2.5 fold increase to TA-P63β (Tables S2, S4 and S5).

Lastly, we examined the relative transactivation specificity of a ΔN-P53 variant *i.e.* Δ40-P53 (obtained from an alternative translation start site) compared to full-length P53. A sample of 10 REs, representative of different P63α isoforms' transactivation specificities, and two different levels of expression of the *GAL1* inducible promoter were used (Figures S2A and B). In all cases, ΔN-P53 exhibited reduced transactivation capacity, with no evidence for a change in transactivation specificity; a C-terminal deletion construct (ΔC-P53), lacking the basic domain was also tested, revealing lower transactivation potential and no impact on specificity (Figures S2A and B).

Overall, the functional analysis in yeast allowed us to establish differences in sequence-specific transactivation for P53 family proteins: i) ΔN- and TA-P63α isoforms (but not the corresponding ΔN- and TA-P63β, P73 or P53 isoforms) exhibited an unexpected difference in relative transactivation specificity towards REs with distinct sequence features; ii) TA-β-isoforms for both P63 and P73 were usually more active than the corresponding α isoforms; iii) regarding the ΔN-β isoforms, ΔN-P63β showed very low levels of activity while ΔN-P73β was virtually inactive.

### Ectopic expression of different P63 isoforms in HCT116 P53^−/−^ cells partially supports the RE-dependent changes in transactivation specificity

We next asked whether the observations in yeast could be confirmed in human cells. For these proof-of-principle experiments, we chose the colon cancer derived HCT116 cell clone where *TP53* has been knocked out and the expression of endogenous P63 or P73 proteins is almost undetectable [39, 40]. Ectopic expression of P63 ΔN, TA, α, β quartet was achieved upon transient transfection. Gene reporter assays, endogenous gene expression (qPCR) and Western blots were used as endpoints, the latter also to control the relative expression of the P63 proteins.

**Figure 3:**
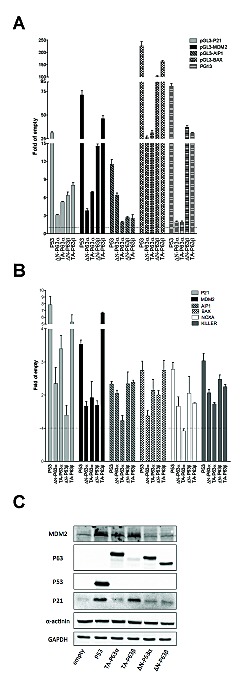
ΔN- and TA-P63α isoforms exhibit some differences in transactivation specificity in HCT116 P53^−/−^ cells A) Dual luciferase assays from HCT116 P53^−/−^ cells transiently cotransfected with the indicated reporter plasmids and P63 expression vectors. P53 was included as control. Renilla luciferase was measured to normalize transfection efficiencies. Data are expressed as fold of induction relative to the results obtained with an empty expression vector. Presented are the average and standard deviations of at least three biological replicates. B) qPCR analysis of the indicated endogenous target genes in HCT116 P53^−/−^ cells transfected with the indicated expression plasmids. Data are normalized to *GAPDH* and *B2M* reference genes and are shown relative to the expression measured from cells transfected with an empty vector. Plots show the average and standard deviations of three technical replicates. The experiment was repeated twice. C) Western Blot analysis of total soluble proteins extracted from HCT116 P53^−/−^ cells transfected with the indicated plasmids. Besides P53 and P63 proteins, the target proteins P21 and MDM2 and the reference controls α-actinin and GAPDH are shown.

With the α isoforms, results with the gene reporter assays confirmed the higher transactivation capacity of TA-P63α with respect to ΔN-P63α on the P21 and MDM2 promoter sequences and the quite similar transactivation on BAX, as highlighted in yeast (Figure 3A). Also the observation that ΔN-P63α was more active than TA-P63α towards the AIP1 reporter was consistent with the results in yeast (Figure 3A). Conversely, P63α isoforms were barely active in HCT116 P53^−/−^ cells towards the PG13 RE cluster, in contrast with what observed in yeast, where both isoforms were active

Endogenous genes expression or Western blot analyses confirmed a higher activity of TA-P63α compared to ΔN-P63α towards P21 and MDM2 (Figure 3B, 3C). Moreover, the expression of ΔN-P63α was also associated with higher endogenous induction of *AIP1* and *NOXA*, as observed in yeast, and the same trend was observed for *KILLER* (Figure 3B). The *BAX* gene was more responsive to TA-P63 than to ΔN-P63α, unlike what obtained in yeast and with the gene reporter assay in mammalian cells. These discrepancies could be due to the known presence of additional REs in the promoter that are not present in the reporter constructs.

Lastly, TA-P63β was the most active isoform also in the human cell line, considering the relative low levels in the transient transfection experiments (Figure 3C). Differently from the yeast results, ΔN-P63β was more active than ΔN-P63α towards the majority of reporter constructs (Figure 3A), although such a difference was not always observed when endogenous genes expression was measured (Figure 3B).

**Figure 4:**
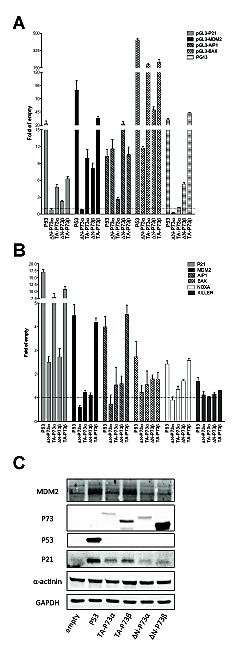
ΔN- and TA-P73 isoforms exhibit differences in transactivation capacity but not in specificity in HCT116 P53-/- cells A) Dual luciferase assays from transiently transfected HCT116 P53^−/−^ cells with the indicated reporter plasmids and P73 isoforms. P53 was included as control. Experiments were performed and data are plotted as described in Figure 3A. B) qPCR analysis of the indicated endogenous target genes in HCT116 P53^−/−^ cells transfected with the indicated expression plasmids. See Figure 3B. C) Western Blot analysis of total soluble proteins extracted from HCT116 P53^−/−^ cells transfected with the indicated plasmids. Besides P53 and P73 proteins, the target proteins P21 and MDM2 and the reference controls α-actinin and GAPDH are shown.

The same experiments were conducted after ectopic expression of P73 quartets (TA, ΔN, α, β) (Figure 4). Results with the α isoforms confirmed those in yeast, showing a higher transactivation capacity of TA-P73s with respect to ΔN-P73s on P21, MDM2 and BAX target sequences. ΔN-P73α was, in fact, inactive towards most reporter constructs (Figure 4A) and endogenous genes tested (Figure 4B). The AIP1 promoter construct, but not the endogenous gene, showed instead higher responsiveness to the ΔN-P73s compared to TA-P73s (α and β isoforms) (Figures 4 A and 4B). ΔN-P73β that was highly expressed (Figure 4C), exhibited a significant activity in the reporter assays (in contrast to what observed with yeast), but was weakly active towards most endogenous genes (Figure 4B), and did not stimulate the expression of P21 and MDM2 proteins (Figure 4C). TA-P73β was in general the most active isoform also in the human cell line.

Overall, the ectopic expression of TA-P63α and ΔN-P63α in HCT116 P53^−/−^ cells partially supports the RE-dependent changes in transactivation specificity.

### Genotoxic stress and/or P53 activating molecules influence the expression of ΔN-P63 isoforms in human cell lines

**Figure 5:**
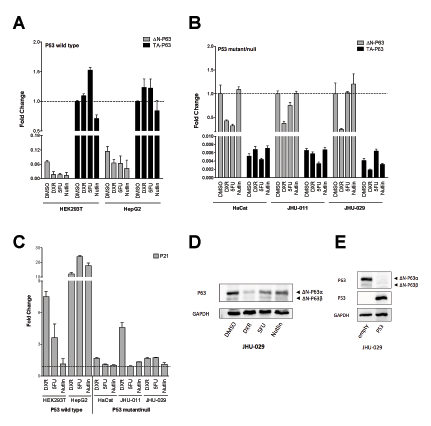
Relative expression of endogenous P63 isoforms in cells lines treated with DNA damaging or P53 inducing agents Experiments were conducted in the indicated cell lines with known P53 status (wild type, mutant or null, as indicated). Cells were treated with Doxorubicin (DXR), 5-FluoroUracil (5FU) or Nutlin-3a (Nutlin) for 16 hours, as described in Materials and Methods. *B2M* and *GAPDH* served as reference genes. Three biological replicates were performed. Measurements of the endogenous levels of ΔN-P63 or TA-P63 mRNAs in P53 wild type A) or mutant/null cells B). For each cell line the graph indicates the relative expression changes and is normalized over the most abundant isoform transcript, set to 1 for the DMSO (mock solvent condition, broken line). C) The endogenous levels of the P53 target gene *P21* were measured as a positive control of the efficacy of the treatments. Values are indicated as fold change relative to DMSO treated cells (set to 1 and indicated by broken line). D) ΔN-P63 protein levels were determined by Western blot in JHU-029 cells after treatment with DXR, 5FU or Nutlin. *GAPDH* was used as loading control. Immunoreactive bands specific for the ΔN-P63α or ΔN-P63β isoforms are indicated with black arrows. E) Western blot experiment demonstrating the over-expression of P53 after transfection and its effect on endogenous ΔN-P63α or ΔN-P63β isoforms in JHU-029 cells. GAPDH was used as loading control.

The relative expression from the P1 and P2 promoters of the *TP63* gene is well-established in the context of squamous epithelial differentiation [41]. Given the identification of a functional p53 RE in the P2 promoter of *TP63* [42-44], we examined whether genotoxic stress or P53 activating molecules could affect the relative expression of P63 promoter variants (TA or ΔN) by qPCR (Figures 5A and 5B). Experiments were performed in five human cell lines that differ for P53 status: HEK293T and HepG2 (wild type), HaCat (mutated), JHU-011 and JHU-029 (null). These cells lines were chosen as they endogenously expressed P63 proteins [45].

In HEK293T and HepG2 cells, ΔN-P63 was expressed at lower levels compared to TA-P63 and its expression decreased both after genotoxic stress or Nutlin treatment, particularly in HEK293T (Figure 5A). TA-P63 levels did not show significant variations except for HEK293T, where a 50% increase and a 25% decrease was observed upon 5FU or Nutlin treatment, respectively (Figure 5A).

In cell lines that are mutated or null for P53, ΔN-P63 was expressed at much higher levels compared to TA-P63 (Figure 5B). DXR led to a marked decrease of ΔN-P63 in all cell lines, while 5FU treatment decreased ΔN-P63 in HaCat and JHU-011 but not in JHU-029; Nutlin did not impact on ΔN-P63 levels in these cell lines (Figure 5B). TA-P63 level was not particularly affected by the treatments, also considering the low expression of this P63 isoform (Figure 5B).

DXR and 5FU led to the induction of the well-known P53 target P21 in P53 wild type cell lines, while Nutlin was effective only in HepG2 cells that do not contain the P53-inhibiting SV40 T-large antigen. These clear expression changes were not detected in the P53 mutated or null cells, with the exception of an induction of P21 in DXR-treated JHU-011 cells.

We selected JHU-029 cells for further studies as they showed the highest endogenous levels of ΔN-P63 through the comparison of the Cycle Threshold in qPCR assays. Consistent with the qPCR results (Figure 5B), also ΔN-P63 protein levels were reduced by genotoxic stress, particularly after the treatment with DXR (Figure 5D). To provide evidence of a direct contribution of P53 in the regulation of endogenous ΔN-P63, we ectopically expressed the wild type protein in JHU-029 cells, which resulted in a severe down-regulation of ΔN-P63 proteins (Figure 5E).

Taken collectively, the results indicate that ΔN-P63 can be negatively regulated at the mRNA and protein levels by DNA damage both in a P53-dependent and -independent manner.

## DISCUSSION

The P53 family of proteins is a paradigmatic group of sequence-specific transcription factors that are master regulators of many different biological responses through the modulation of a very large number of target genes. While genetic approaches indicate a clear separation of functions acquired after the gene duplication events throughout evolution that led to the three-genes *TP53* family, biochemical and molecular biology approaches, including genome level occupancy and transcriptome analyses, indicate a high degree of conservation of the core function, i.e. the sequence-specific DNA binding [10, 27, 46-51]. Another contribution to the complexity of this scenario is represented by the modulation of these transcription factors in the context of acute stress conditions, such as DNA damage, that can impact on transactivation selectivity *in vivo*. These layers of regulation have been studied more in-depth for P53, for which post-translational modifications affecting protein stability, localization and interactions, along with the contribution of context dependent *trans*-factors, are recognized elements that modulate transcriptional specificity [52-54]. The contribution of *cis-*factors, namely specific features of the target DNA sites, here referred as p53 REs, has also been extensively studied [52, 53]. Less is known about the regulation of P63 and P73, although DNA damage is a recognized activation stimulus and specific post-translational modifications have been identified [55-57]. Recently, several studies have uncovered that cooperative DNA binding coupled to RE sequence differences at specific target genes can modulate P53 transactivation selectivity [28, 58-60]. For example, *TP53* mutations or post-translational protein acetylation were associated to changes in cooperative DNA binding and to a selective modulation of apoptotic target genes [61]. Also, biophysical measurements led to classify p53 REs in terms of low or high cooperativity; kinetic features, especially off-rates, correlate with transactivation potential more than DNA binding affinity measured with purified P53 DBDs at low protein concentrations [30].

To establish the role of RE sequence in transactivation specificity of P63 promoter- (ΔN and TA) and splice-variants (α and β), an experimentally defined assay in yeast was used, where the sequence of the RE under study and the expression of a chosen P63 isoform are the only variable and quantitative luciferase reporter assays are the functional endpoint. Our results revealed that ΔN-P63α and TA-P63α not only differ in transactivation potential, but also in transactivation specificity (Figure 1A-D; Table S1A, S1B). By using a large number of REs, we were able to highlight sequence features that correlated with the changes in transactivation specificity. In particular, ΔN-P63α-preferred REs were associated with CWWG core motifs characterized by 1) a lower torsional flexibility, relative to generic B-DNA sequences, as determined from cyclization kinetics experiments, [30, 38], 2) higher number of mismatches (Table S1A), and 3) in some cases, presence of a short spacer among decameric half-sites or absence of one pentameric quarter site (Table S1B). Hence, ΔN-P63α, compared to TA-P63α, exhibited an intrinsic preference for lower affinity (Figure 1E and 1F), high cooperativity target sequences. A possible explanation for the observed differences is that the preferred quaternary structure would be different for these two P63 isoforms, with ΔN-P63α, but not TA-P63α, being a tetramer. Notably, it was recently reported that P63 isoforms in solution can adopt different quaternary structures and specifically that TA-P63α, unlike ΔN-P63α, would not form tetramers due to an intramolecular inhibiting interaction [8, 62]. Interestingly, DNA damage-induced post-translational changes can lead to TA-P63α tetramer formation [8, 63]. In this regard, ΔN-P63α could be considered as a constitutively active tetramer. Our functional results in yeast are consistent with those data, in that a tetrameric conformation is expected to retain the capacity to interact with REs that, due to mismatches or non-canonical sequence features, would not efficiently enable independent binding of two protein dimers. The lack of the entire TA1 transactivation domain in ΔN-P63α led, however, to a lower extent of reporter genes induction, evident for the REs where both ΔN- and TA-P63α were active (Table S1). Also consistent with this interpretation are our recent results with P63 mutations associated to ectodermal dyplasia syndromes that exhibited a more severe defect when expressed in yeast as TA-P63α compared to ΔN-P63α isoform (Figure S3) [39].

Importantly, ectopic expression of ΔN- and TA-P63α in an otherwise untreated HCT116 cell clone, that is null for P53 and expresses negligible levels of endogenous P63 or P73 (9,46), confirmed the differences in transactivation specificity using gene reporter assays and, in part, also following modulation of endogenous genes. Previous data in mammalian systems had also observed higher activity of ΔN-P63α compared to TA-P63α for selected target genes [11, 64]. Hence, the specific responsiveness of the REs can, to some extent, impact on the relative expression changes even when the RE is embedded in the natural promoter context. However, yeast-based assays and also results with cloned promoter fragments in mammalian cells were not completely predictive of the responsiveness of endogenous target genes, in particular when multiple REs are present in the regulatory regions, such as in the case of *BAX*.

Our results regarding the ΔN-P63β isoform are strikingly inconsistent between yeast and human cells: in fact in yeast ΔN-P63β showed much lower transactivation capacity than ΔN-P63α (Figure 2A and Table S2) (Figure 3). The low activity of ΔN-P63β in yeast did not appear to be caused by reduced protein expression (Figure S1), but we cannot exclude reduced affinity for components of the transcription machinery, or lack of necessary cofactors, or even a defect in nuclear import. Conversely, in HCT116 P53^−/−^ cells, ΔN-P63β was much more active than ΔN-P63α and in some cases equal to TA-P63β, but without a clear evidence for changes in transactivation specificity (Figure 3). The results with P63β isoforms in HCT116 and with TA-P63β in yeast are also consistent with the reported tetrameric nature of P63β variants in solution [8, 65]. Furthermore, the comparison between TA-P63α and TA-P63β in yeast and in mammalian cells evidenced the higher transactivation potential of the β splice variant lacking the SAM domain and the carboxy-terminal inhibitory domain as well as the capacity of TA-P63β to retain activity towards REs containing spacers or non-canonical three-quarter site REs. This feature was particularly evident in HCT116 cells with the PG13 RE, consisting of a tandem repeat of 13 copies of a low affinity RE of 19 nucleotides.

We also studied TA- and ΔN-, α and β variants of P73. In the yeast system ΔN-P73α was an extremely weak transcription factor and ΔN-P73β was inactive (Figure 2B and Table S3). In HCT116 P53^−/−^, although results might be biased by the higher protein expression, ΔN-P73β exhibited activity with all reporters tested and was even more active than TA-P73β with the AIP1 and BAX promoter constructs (Figure 4). However, ectopic expression of both ΔN-P73α and ΔN-P73β did not lead to the induction of most of the endogenous target genes tested. Further studies are needed to examine more precisely the possibility that promoter and/or splice variants of P73 would exhibit differences in transactivation specificity and to elucidate the potential for ΔN-P73s to modulate gene expression in a physiological setting. TA-P73α has been reported to be tetrameric by size exclusion chromatography, suggesting a closer similarity with P53 than with TA-P63α in stress-induced regulation of transcriptional activity [65].

All P63 and P73 isoforms tested in yeast were inactive on half-site REs (Tables S2, S3 and S4). When expressed at high levels both in yeast and in human cells, P53 was instead shown to be capable of modulating transcription from half-site REs as well as to bind to half-site REs by ChIP and ChIP-sequencing experiments [66, 67]. However, the oligomeric state of P53 protein bound to half-sites remains to be established.

Several P53 isoforms have also been identified, resulting from alternative splicing of intron 2 or alternative translation initiation (ΔN-P53, ΔC-P53), an internal promoter (P53-Δ133), or alternative splicing at the carboxy terminus (P53β, P53γ) [68]. Of these isoforms, only ΔN-P53 retains the entire DBD and oligomerization domains [47]. Very recently, a mouse model with homozygous deletion of the basic domain in the P53 C-ter was reported to have severe phenotypes, very reminiscent of Dyskeratosis congenita [69]. In derived MEFs (mouse embryonic fibroblasts), constitutive nuclear levels of this C-ter deleted-P53 were slightly higher compared to wild type controls, but the possibility of changes also at the level of relative transactivation specificity was not conclusively investigated. An independent mouse model of the same P53 C-ter deletion construct reported tissue-specific differences on the transactivation of target genes [70]. Those findings along with data obtained with P63α isoforms and on the oligomeric state of P53 family proteins [65], stimulated us to examine P53 N-ter (ΔN-P53) and C-ter deletion (ΔC-P53) constructs using the yeast assay in order to investigate possible changes in transactivation specificity. The results obtained with ten selected REs showed that both P53 deletion constructs retained some level of transactivation potential but they were less active than wild type P53 and did not exhibit differences in specificity (Figure S2). However, the possibility of RE-driven allosteric changes that are not impacting on transactivation in yeast due to missing cofactors cannot be entirely excluded.

To explore the potential for evolutionary conservation of transactivation capacity and specificity of P63 proteins, ΔN-P63 from zebrafish (Danio rerio, Dr), an organism where the P2 promoter could be predominantly or exclusively active [71] was cloned and tested in yeast. Moreover, phylogenetic studies have not conclusively demonstrated which TP63 promoter is more ancient between P1 (TA) and P2 (ΔN) [71, 72]. We were able to clone a single ΔN-P63 isoform, corresponding to ΔN-P63β, and considered the main or the exclusive isoform expressed in zebrafish [71]. Nevertheless, the protein was an active transcription factor in yeast. A comparison of Dr-ΔN-P63 with human TA-P63α indicated an overall conservation of relative transactivation specificity as seen for human ΔN-P63α in the comparison with TA-P63α (Figure S4 and Table S1).This observation may suggest that Dr ΔN-P63, like human ΔN-P63, constitutively adopts a tetrameric conformation.

Alternative *TP63* promoter usage in mammals has been clearly established as part of differentiation programs, notably in the context of squamous cell epithelia, where basal layer cells express predominantly ΔN-P63, while TA-P63 expression is prevalent in the differentiated cells above [41]. We were interested in exploring whether changes in relative promoter activity could also occur in the context of DNA damage, also in the light of the established presence of a p53 RE in the *TP63* promoter P2 [42-44]. Interestingly, P53 has also been reported to modulate the activity of the *TP73 P2* promoter [73] [42]. Experiments in five cell lines expressing endogenously TA- or ΔN-P63 and differing for P53 status indicated that genotoxic stress, or P53 activation with Nutlin in P53 wild type cells, or P53 ectopic expression in a P53 null cell line, can lead to a decrease in ΔN-P63 protein levels, thereby changing the ratio between TA and ΔN isoforms (Figure 5). Given the different transactivation specificities and biological functions of ΔN- and TA-P63 these changes in relative expression might influence the cellular response by modulation of expression of target genes.

Our results indicate that the regulation of P63 promoters' usage could affect P63-dependent transactivation specificity, exploiting the differences in oligomeric state between ΔN- and TA-P63α [65] and not simply tune the levels of target gene expression.This regulatory mechanism would however require a co-evolution of promoter RE features among different functional groups of P63 target genes to be able to impact on biological outcomes. The apparent enrichment for low affinity/high cooperativity REs among established P53 family target genes involved in apoptosis [30, 58, 59, 61] could reflect this coevolution and be interpreted in the light of the results establishing the ancestral function of P63-like proteins as the quality control of the germline via induction of apoptosis.

## MATERIALS AND METHODS

### Yeast strains, vectors and functional assay

For analysis of the transactivation ability of P53 family members (P53, ΔN- and TA-, -α and -β variants of P63 and P73) we used 87 haploid *S. cerevisiae* yeast strains (yLM-REs) [28, 34-36, 45, 74]. All strains are isogenic except for the different RE located upstream of the luciferase reporter gene [29, 36, 66, 75]; new yLFM-RE strains were constructed, taking advantage of the *Delitto perfetto* approach [76, 77]. The panel of REs includes 53 canonical REs (20 bp) without spacer (Table S1A), 15 canonical REs with a spacer and 19 non-canonical REs comprising three-quarter sites, half-sites, multimers and altered-structure REs (Table S1B).

Yeast manipulations were performed as previously described [28, 34, 39]. Inducible expression of P53 family members was achieved under a *GAL1-10* promoter using a pTSG-based (TRP1) vector. Vectors expressing human ΔN-P63α, TA-P63α and P53 were already available [29, 31, 39]. New pTSG-based vectors expressing human Δ40-P53, ΔC-P53 (Δ369-393), ΔN-P63β, TA-P63β, ΔN-P73α, TA-P73α, ΔN-P73β and TA-P73β were constructed as previously described [39]. ΔN-P63β and TA-P63β cDNAs were provided by Prof. Roberto Ravazzolo and Dr. Renata Bocciardi (IRCCS G. Gaslini, Genoa, Italy). pTSG-based vectors expressing *TP63* mutants as ΔN-P63α isoform were already available, while the same mutants as TA-P63α variants were constructed as previously described for the pTSG-TA-P63α vector (43). A pTSG vector harboring the ΔN-P63β isoform expressed in zebrafish (*Danio rerio*) was also constructed. Plasmid pRS314 (TRP1) was used as empty vector. Primer sequences are available upon request.

The transactivation ability of the different P53 family variants was analyzed by transforming yLFM-REs strains with the different expression vectors using the Lithium acetate method, as previously described [34]. The luciferase assay was conducted according to the miniaturized protocol we recently developed [34]. Yeast transformants were grown (8 or 16 hours as indicated) in media containing raffinose (2%) without or with different concentrations of galactose as inducer. The transactivation ability of P53 family members was measured using the Bright Glo Luciferase assay kit (Promega, Milan, Italy) and expressed as relative light unit (RLU) normalized to optical density (600 nm), subtracting the luminescence obtained by the cells transformed with the empty vector in each reporter strain.

### Mammalian cell lines and vectors

Human colon cancer HCT116 P53^−/−^ cell line was a gift of Prof. B. Vogelstein (John Hopkins University, Baltimore, MD, USA) and was used for transfecting constructs expressing P53 family members, followed by gene reporter assays, quantitative PCR and Western blotting experiments. HepG2 (derived from a human hepatocellular carcinoma and wild-type at the *TP53* locus) and HEK293T (derived from human embryonic kidney and wild-type at the *TP53* locus, but expressing the P53-inhibiting SV40 T-large antigen) were obtained from Dr. Alessandro Provenzani (CIBIO, University of Trento, Italy) and Prof. Juergen Borlak (Hannover Medical School, Germany), respectively. HaCat cells (human immortalized keratinocytes harboring the compound heterozygous *TP53* mutations H179Y and R282W) as well as the P53-null JHU-011 and JHU-029 cells (Head and Neck Squamous Cell Carcinoma-derived human cell lines) were obtained from Prof. David Sidransky's laboratory at John Hopkins University (Baltimore, MD, USA). HepG2, HEK293T, HaCat, JHU-011 and JHU-029 were used to test the effect of P53 activating molecules on P63 isoforms expression levels. Cells were cultured in DMEM or RPMI (GIBCO, Invitrogen, Milan, Italy) supplemented with 10% fetal bovine serum (FBS), 2mM L-Glutamine and antibiotics (100 units/ml penicillin plus 100 μg/ml streptomycin) and maintained in a humidified atmosphere at 37°C with 5% CO_2_. Cells were routinely checked to exclude the presence of mycoplasm.

pCI-neo plasmids for the expression of ΔN-P63α and TA-P63α were already available [39]. pCI-neo plasmids expressing ΔN-P63β, TA-P63β, ΔN-P73α, TA-P73α, ΔN-P73β and TA-P73β were obtained by XhoI/NotI double digestion of pTSG-vectors containing the desired cDNAs and subsequent ligation of the insert into equally digested pCI-neo plasmid. P53 expression was obtained using a pCI-neo-derived plasmid similarly generated. The empty pCI-neo plasmid was used as control vector.

The pGL3-P21 (2.3 Kb promoter fragment, containing both the 5' and the 3' p53 REs of the *P21* gene), pGL3-MDM2 (350 bp region with both p53 REs present in intron 1 of the *MDM2* gene), PGL3-BAX (400 bp region of intron 1 of the *BAX* gene), PG13 (13 direct repeats of the sequence 5'-CCAGGCAAGTCCAGGCAGG-3') and pGL3-AIP1 (containing the p53 RE from yFLM-AIP1 yeast strain) reporter vectors were used for luciferase reporter assay in mammalian cells after transient transfection [28]. pRL-SV40 plasmid, harboring the luciferase gene from *Renilla reniformis* under the control of a constitutive promoter, was used to normalize for transfection efficiency.

### Luciferase assay in the mammalian cell line HCT116 P53-/-

HCT116 P53^−/−^ cells (2x10^5^) were seeded twenty-four hours prior transfection onto 24-well plates. Cells were co-transfected at 50-70% confluence using TransIT-LT1 (Mirus, Tema Ricerca, Bologna, Italy) with: i) 200 ng of the expression vectors (for P53 expression, 50 ng of vector plus 150 ng of empty vector were used -200 ng total DNA amount- in order to avoid excessive toxicity); ii) 250 ng of the reporter vector and iii) 50 ng of the normalization vector. After additional 24 hours, cells were washed with PBS, lysed with Passive Lysis Buffer (PLB) 1X (Promega). Firefly and Renilla luminescence were measured as described previously [78].

### Transfections and treatments with P53 inducing/activating compounds

HCT116 P53^−/−^ and JHU-029 cells were seeded onto 6 well plates at the concentration of 9x10^5^ cells per well and transfected the following day with 2 μg of expression vectors for P53 family proteins (in pCI-neo) or pCI-neo empty vector, using Fugene HD (Promega). Twenty four hours later, for transfections, or when indicated, for treatments, cells were harvested and processed for RNA or protein extraction.

P53 wild-type (HEK293T and HepG2) as well as P53 mutated (HaCat) or P53 null (JHU-011 and JHU-029) cell lines that endogenously express P63 were treated with compounds able to stabilize and activate P53. Twenty-four hours after seeding, cells were treated with Doxorubicin (DXR -1.5μM), 5-FluoroUracil (5FU -375μM) and Nutlin-3A (Nutlin -10μM) for 16 hours and then processed as indicated above. All compounds were purchased from Sigma Aldrich (Milan, Italy). DMSO was used as control as 5FU and Nutlin were dissolved in such solvent.

### Quantitative PCR analyses

To determine optimal transfection efficiency pEGFP-N1 vector was used (obtained from Prof. Paolo Macchi, CIBIO, University of Trento). Twenty-four hours post-transfection cells were harvested and total RNA was isolated using the RNeasy mini kit, according to the manufacturers' recommendations (Qiagen, Milan, Italy). Two μg of RNA were converted into cDNA using the M-MuLV reverse transcriptase (Thermo Scientific, Milan, Italy). Quantitative PCR was performed on 25 ng of cDNA as previously described [79] using KAPA Sybr Green Master mix (Kapa Biosystems, Resnova, Ancona, Italy) and specific primers to measure the expression of *TP63* (ΔN and TA), *P21, MDM2, AIP1, BAX, KILLER* and *NOXA* genes. *B2M* (β2-microgobulin) and *GAPDH* (Glyceraldehyde 3-Phosphate DeHydrogenase) were used as reference genes. Primer sequences are available upon request. Fold changes respect to the empty vector were calculated using the ΔΔC_T_ method [80].

### Antibodies and Western blotting

#### Protein extraction from yeast cells

Yeast transformants were grown for 8 hours in selective medium containing 0.128% galactose to induce the expression of P63 and P73 specific isoforms. An equivalent amount of cells, based on the culture absorbance measurement (2.5 OD, OD_600nm_), was collected by centrifugation. Cells were treated with 0.2N NaOH at room temperature following the extraction protocol described in [81] and 25 μl of extracts were loaded on 7.5% poly-acrylamide gels. Transfer onto nitrocellulose membranes was achieved using the i-Blot semi-dry system (InVitrogen, Life Technologies, Milan, Italy). Specific antibodies directed against P63 (clone 4A4: sc-8431, Santa Cruz Biotechnology, Milan, Italy) or P73 isoforms (clone ER-15: OP109, Calbiochem, Millipore, Milan, Italy), were diluted in 1% non-fat skim milk dissolved in PBS-T. PGK1 (Phospho Glycerate Kinase 1) was used as loading control (clone 22C5D8, Life Technologies, Milan, Italy).

#### Protein extraction from mammalian cells

Pellets from transfected or treated cells were washed with PBS and used for total protein extraction in RIPA lysis buffer supplemented with Protease Inhibitors cocktail (Roche, Milan, Italy). Besides the antibodies against P63 and P73 described above, antibodies against P53 (clone DO-1: sc-126), P21 (clone C-19: sc-397), MDM2 (clone SMP-14: sc-965), GAPDH (clone 6C5: sc-32233) or α-actinin (clone H-2: sc-17829) (all from Santa Cruz Biotechnology) were used for immunodetection after dilution in 1% non-fat skimmed milk dissolved in PBS-T. Immuno-reactive bands from yeast as well as mammalian extracts were detected by the ChemiDoc XRS+ System (BioRad, Milan, Italy), using the ECL Select detection reagent (Amersham, GE Health Care, Milan, Italy).

## SUPPLEMENTARY FIGURES AND TABLES




