# Supplementary material for: ΔN-P63α and TA-P63α exhibit intrinsic differences in transactivation specificities that depend on distinct features of DNA target sites
Source: Oncotarget. 2014 Mar 23;5(8):2116–30. doi: 10.18632/oncotarget.1845 (PMC4039150; doi:10.18632/oncotarget.1845)
Supplement: Supplementary file 1 [file oncotarget-05-2116-s001.pdf]

ΔN-P63α and TA-P63α exhibit intrinsic differences in transactivation specificities that depend on distinct features of DNA target sites – Monti et al

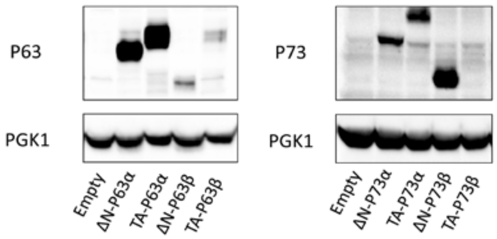

Figure S1**. Western blot analyses of P63 and P73 expression in yeast.**

Yeast cells transformed with the indicated plasmids were cultured for 8 hours in medium containing galactose (0.128%). Cells were collected and treated as described in Materials and Methods and soluble extracts used for western blotting and immunodetection with anti-P63 (A) and P73 (B) antibodies. An antibody specific to yeast PGK1 was used as loading control.

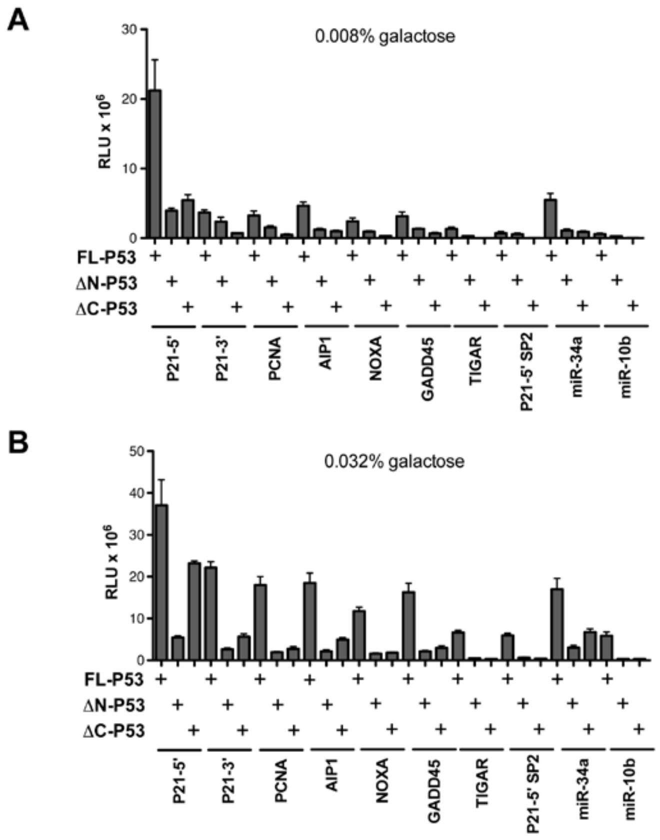

Figure S2. **ΔN- and ΔC-P53 proteins do not exhibit differences in relative transactivation specificity**.

A P53 deletion construct lacking the first 39 amino acids (ΔN-P53), corresponding to the Δ40 isoform and a deletion construct lacking the last 25 amino acids, containing the basic domain (ΔC-P53), were expressed in yeast and tested for transactivation activity using 10 different REs. The full-length wild type P53 was included as a control. P53 proteins were expressed under an inducible *GAL1,10* promoter. Results were obtained at low (**A**, 0.008% galactose) or moderate (**B**, 0.032% galactose) expression levels after 8 hours of growth. Presented in the bar graphs are average luciferase activity and standard errors of four biological replicates. Luciferase light units (RLU x 10^6) were normalized to the optical density of the cultures. The P53-independent reporter activity, measured from

each reporter strains transformed with an empty vector, was also subtracted for normalization.

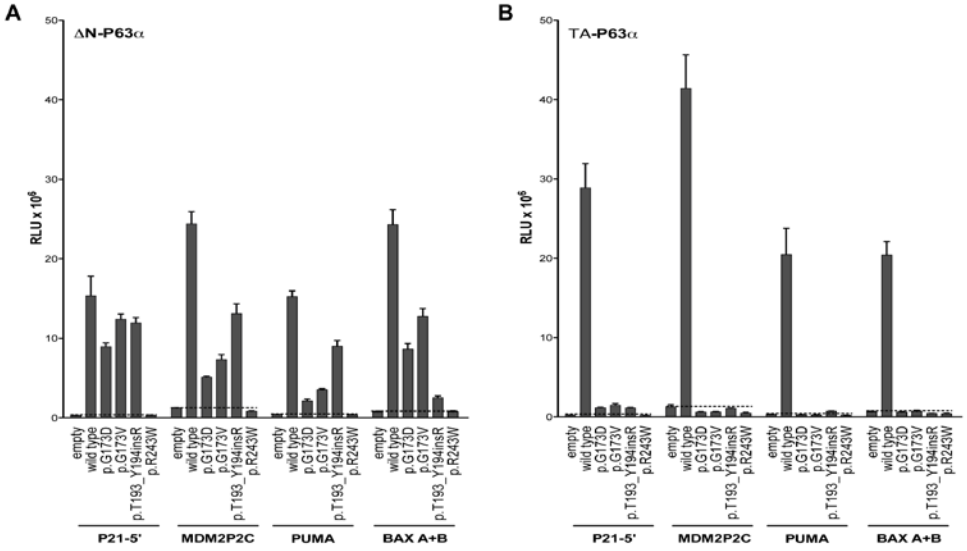

Figure S3. **P63 mutations associated to ectodermal dysplasia syndromes exhibit a more severe transactivation defect when expressed in yeast as TA-P63α compared to DN-P63α isoforms**. Transactivation ability of wild type and mutant proteins of P63 in yLFM-P21-5’, yLFM-MDM2, yLFM-PUMA and yLFM-BAX yeast strains. The transactivation ability was determined as in Figure 1. ΔN-P63α and TA-P63α isoforms were shown in panel A and B, respectively. Values obtained with an empty vector (pRS314) were also reported and indicated by a broken line. Results are presented as average RLU with standard deviations.

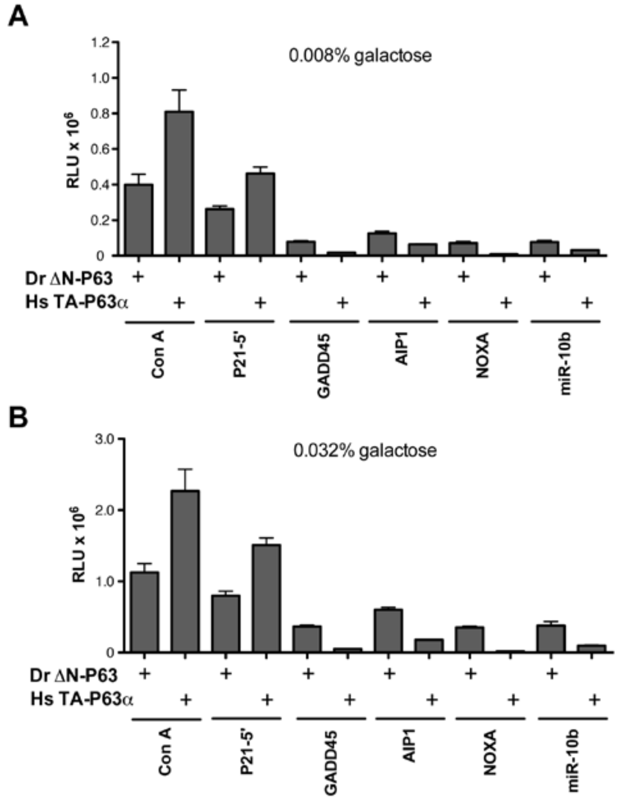

Figure S4. **The transactivation specificity of human ΔN-P63α is conserved in *Danio rerio* ΔN-P63**.

*Danio rerio (*Dr*)* ΔN-P63 cDNA was cloned into a yeast expression vector under the inducible *GAL1,10* promoter. The induction of the *Firefly* Luciferase reporter was measured after 16 hours fo growth for the indicated reporter strains transformed with an empty vector (used for normalization), a vector expressing Dr P63, or a vector expressing human TA-P63α that was included to enable comparisons with the data presented in Table S1A and S2. Luciferase activity was normalized to cell density (absorbance at OD_{600nm}). Presented are the average and standard errors of four biological replicates. The experiment was conducted by inducing expression of the proteins at two different levels of galactose and after 8 hours of growth.

REFERENCES

1. Andreotti V, Ciribilli Y, Monti P, Bisio A, Lion M, Jordan J, Fronza G, Menichini P, Resnick MA and Inga A. p53 transactivation and the impact of mutations, cofactors and small molecules using a simplified yeast-based screening system. PLoS ONE. 2011; 6(6):e20643.
