# Supplementary material for: ΔN-P63α and TA-P63α exhibit intrinsic differences in transactivation specificities that depend on distinct features of DNA target sites
Source: Oncotarget. 2014 Mar 23;5(8):2116–30. doi: 10.18632/oncotarget.1845 (PMC4039150; doi:10.18632/oncotarget.1845)
Supplement: Supplementary file 2 [file oncotarget-05-2116-s002.pdf]

ΔN-P63α and TA-P63α exhibit intrinsic differences in transactivation specificities that depend on distinct features of DNA target sites – Monti et al

Table S1 A and B. Relative transactivation capacity and specificity of all REs tested with ΔN-P63α and TA-P63α using the yeast-based transactivation assay.

Summary of the ΔN-P63α and TA-P63α transactivation capacities towards 87 REs measured using the yeast functional assay. For all the experiments the RLU was normalized on cell number measured by optical density OD_{600} [1] and subtracted for the value obtained with transformants containing an empty expression vector (pRS314). P63α isoforms were expressed under the *GAL1* inducible promoter (8 hours at 0.128% galactose). The REs are ordered based on increasing ΔN-P63α/TA-P63α ratio. The columns of the table report: 1) the name of the RE, grouped for different canonical and non-canonical categories; 2) the sequence of the RE (the CORE sequence is indicated in bold; mismatches with respect to the consensus sequence are indicated in red); 3) the transactivation ability (as Relative Light Unit: RLU x 10^5) of ΔN-P63α; 4) the transactivation ability (RLU x 10^5) of TA-P63α; 5) the ΔN-P63α/TA-P63α transactivation ratio; 6) the total number of mismatches of the RE sequence; 7) the predicted binding affinities (Log K_d). The canonical REs (i.e. REs consisting of two decameric half sites without spacer) were used to compile the Logos in Figure 1C and 1D. These were grouped as TA-P63α–preferred REs when the ΔN-P63α/TA-P63α transactivation ratio was lower than 0.67 (<-0.17 in log scale; i.e. TA-P63α activity was at least 1.5 times higher than TA-P63α activity) or as ΔN-P63α–preferred REs when the ΔN-P63α/TA-P63α transactivation ratio was greater than 1.5 (>0.17 in log scale; *i.e.* ΔN-P63α transactivation activity at least 1.5 times higher TA-P63α activity). The TA-P63α– and ΔN-P63α–preferred REs are highlighted in gray and light blue, respectively. The ratio between ΔN-P63α/TA-P63α activities was not assessable (NA) when either protein was inactive (ND, not detectable) towards a given RE. These REs are highlighted in green. Canonical REs exhibiting a ΔN-P63α/TA-P63α transactivation ratio comprised between the two cut-offs (>0.67 and <1.5) are not highlighted and were excluded from the Logo summary view.

Canonical without spacer	RRRCWWGYYY-RRRCWWGYYY	ΔN-P63α (RLU x 10^5)	TA-P63α (RLU x 10^5)	ΔN-P63α /TA-P63α	Total No. mismatches	Log_{10} K_d
C3 SP0 SNP	GGG**CATG**TCC-GGA**CATG**TCC	102.38	390.56	0.26	0	-7.51
C2 SP0	GGG**CATG**CCC-GGG**CATG**CCC	128.93	471.94	0.27	0	-7.61
CON A	GGG**CATG**TCC-GGG**CATG**TCC	129.07	453.34	0.28	0	-7.56
CON P	GGG**CATG**TCC-GAG**CATG**TCC	117.65	367.78	0.32	0	-7.46
H1	GGG**CATG**TTC-GAA**CATG**TCC	141.78	395.47	0.36	0	-7.31
CON G	GGG**CATG**TCT-GGG**CATG**TCT	172.44	473.80	0.36	0	-7.48
H1 SNP	GGG**CATG**TTC-GAA**CATG**TTC	99.74	239.03	0.42	0	-7.28
CON Q	GGG**CATG**TCT-GGG**CAAG**TCT	106.23	244.35	0.43	0	-7.27
CON L	GGG**CATG**CTC-GGG**CATG**CTC	159.21	364.92	0.44	0	-7.48
R2	TGA**CATG**CCC-AGG**CATG**TCT	134.09	293.33	0.46	1	-7.46
P21-5'	CAA**CATG**TTG-GGA**CATG**TTC	128.94	277.19	0.47	2	-7.11
CON C	GGG**CAAG**TCT-GGG**CAAG**TCT	183.20	363.79	0.50	0	-7.11
CON E	GAG**CATG**TCC-GAG**CATG**TCC	148.77	288.23	0.52	0	-7.43
PUMA	CTG**CAAG**TCC-TGA**CTTG**TCC	134.84	206.54	0.65	3	-6.71
PA26	GGA**CAAG**TCT-CAA**CAAG**TTC	126.55	175.42	0.72	1	-6.85
C1 SP0 SNP	GGG**CATG**CCT-AGG**CATG**CCT	109.02	146.80	0.74	0	-7.5
m-FAS	GGG**CATG**TAC-AAA**CATG**TCA	204.86	250.96	0.82	2	-7.16
C3 SP0	GGA**CATG**TCC-GGA**CATG**TCC	130.88	135.12	0.97	0	-7.51
MMP2	AGA**CAAG**CCT-GAA**CTTG**TCT	114.72	103.12	1.11	0	-7.01
miR-34a RE1	GGG**CTTG**CCT-GGG**CTTG**TTC	152.47	131.28	1.16	0	-7.18
miR-198 SNP	AGG**CATG**CTT-CAA**CATG**CCG	99.56	84.10	1.18	2	-7.04
KILLER	GGG**CATG**TCC-GGG**CAAG**ACG	144.60	119.04	1.21	2	-7.09
h-FAS	TGG**CTTG**TCA-GGG**CTTG**TCC	126.43	99.80	1.27	2	-7
C1 SP0	AGG**CATG**CCT-AGG**CATG**CCT	176.77	119.02	1.49	0	-7.45
P21 S2	GAA**CAGG**TCC-CAA**CAGG**TTG	191.19	123.18	1.55	4	-6.29
miR-202	GGG**CATG**TCT-TGG**CAAG**CCT	72.49	44.48	1.63	1	-7.15
miR-151	TGG**CTTG**TTT-TGG**CAAG**TTC	30.27	10.77	2.81	2	-6.79

**AIP1**	TCTCTTGCCC-GGGCTTGTCG	130.38	46.23	2.82	4	-6.48
**CYCLIN G**	AGGCTTGCCC-GGGCAGGTCT	109.47	31.11	3.52	0	-6.83
**CON B**	GGGCTAGTCC-GGGCTAGTCC	14.71	3.10	4.74	0	-6.82
**GADD45**	GAACATGTCT-AAGCATGCTG	121.60	25.42	4.78	1	-7.23
**PRODH +6.8**	AGGCTTGCCT-CAGCATGTCG	140.01	22.68	6.17	2	-6.98
**PERP**	AGGCAAGCTC-CAGCTTGTTC	99.92	15.96	6.26	1	-6.88
**miR-221**	GAACATGCAT-GCACATGTTT	122.35	18.36	6.66	2	-6.98
**miR-198**	AGGCAAGCTT-CAACAAGCCG	96.24	11.47	8.39	2	-6.67
**COL18A1 ISOFORM2***	AGACATGCAC-ACACATGCAC	56.72	4.34	13.08	3	-6.94
**PAI**	ACACATGCCT-CAGCAAGTCC	74.40	5.62	13.24	2	-6.51
**NOXA**	AGGCTTGCCC-CGGCAAGTTG	96.55	7.14	13.52	2	-6.87
**COL18A1 ISOFORM1 SNP1***	GGGCATGCTG-GGGCATGGCA	125.92	8.72	14.44	3	-6.91
**PCNA**	GAACAAGTCC-GGGCATATGT	97.18	5.93	16.39	1	-6.83
**H2**	GAACTAGTTC-GAACTAGTTC	43.15	1.03	41.82	0	-6.51
**XPC**	GGGCATGGTG-GCACATGCCT	50.96	1.13	45.21	3	-6.52
**PRODH +1.7**	GGGCAAGGAC-GGGCATGCTA	18.60	0.11	161.82	3	-6.73
**P48**	AAGCTGGTTT-GAACAAGCCC	15.12	ND	>161.82	1	-6.43
**COL18A1 ISOFORM1***	GCACATGCTG-GGGCATGGCA	33.00	ND	>161.82	4	-6.33
**ZAC1 SNP**	CAACTAGACT-AGACTAGCTA	0.16	ND	NA	3	-6.27
**KRT14**	AGGCAGCCCC-AGACACGATC	0.20	ND	NA	3	-6.06
**ZAC1**	CAACTAGACT-AGACTAGCTT	0.52	ND	NA	2	-6.29
**JAG2**	ACCCATGCCC-GTCCACGCTC	1.32	ND	NA	5	-6.05
**DLX6**	GAGAATAGCC-AGGCATGTAG	ND	0.17	NA	4	-5.73
**KRT14 SNP**	GGGCAGCCCC-AGACACGATC	ND	2.78	NA	4	-6.11
**CaN19**	CACCATGCCT-GGCCAATTTT	ND	ND	NA	4	-6.09
**a-1AT**	TCCCATGGCT-AGGCATGAGA	ND	ND	NA	7	-6.03

* indicated as COL18A1 I2, COL18A1 I1 SNP1, and COL18A1 I2 in Figure 1A

Table S1B

**Canonical with spacer**	**RRRCWWGYYY-n-RRRCWWGYYY**	**ΔN-P63α (RLU x 10^5)**	**TA-P63α (RLU x 10^5)**	**ΔN-P63α / TA-P63α**	**Total No. mismatches**
C2 SP1	GGG**CATG**CCC-c-GGG**CATG**CCC	86.51	8.85	9.77	0
C2 SP4	GGG**CATG**CCC-gcgc-GGG**CATG**CCC	71.01	3.49	20.32	0
C1 SP4	AGG**CATG**CCT-gcgc-AGG**CATG**CCT	54.32	1.95	27.85	0
PRODH +4.7	GTC**CTTG**TTG-cca-GGG**CATG**CCT	16.59	ND	>27.85	3
H3	GAA**CATG**TTC-g-GAA**CATG**TTC	25.54	ND	>27.85	0
C2 SP2	GGG**CATG**CCC-gc-GGG**CATG**CCC	74.29	ND	>27.85	0
PRODH -3.1	CGA**CTTG**TCC-tcaat-GAC**CAC**GCTC	0.71	ND	NA	3
C1 SP2	AGG**CATG**CCT-gc-AGG**CATG**CCT	0.70	0.02	NA	0
C3 SP2	GGA**CATG**TCC-at-GGA**CATG**TCC	ND	ND	NA	0
C3 SP4	GGA**CATG**TCC-atat-GGA**CATG**TCC	ND	ND	NA	0
P21-5' SP2	CAA**CATG**TTG-gc-GGA**CATG**TTC	0.11	0.31	NA	2
P21-5' SP5	CAA**CATG**TTG-ggcgt-GGA**CATG**TTC	0.19	ND	NA	2
CON B SP2	GGG**CTAG**TCC-gc-GGG**CTAG**TCC	0.15	0.06	NA	0
CON B SP4	GGG**CTAG**TCC-tgct-GGG**CTAG**TCC	0.04	0.56	NA	0
CON A SP4	GGG**CATG**TCC-tgct-GGG**CATG**TCC	0.24	ND	NA	0
**Non-canonical three-quarter site**	**XXXXX-RRRCWWGYYY-XXXXX**	**ΔN-P63α (RLU x 10^5)**	**TA-P63α (RLU x 10^5)**	**ΔN-P63α /TA-P63α**	**Total No. mismatches**
miR-10b	TGTCT-GAA**CAAG**TCG	103.13	41.23	2.50	1
CON J	GGG**CATG**TCC-GGGCAccaca	72.25	10.93	6.61	0
P21-3' Complete	GGG**CATG**TCT-GGGCAc	67.57	6.84	9.88	0
CON K	GGG**CATG**TCC-tgtttTGTCC	37.79	ND	>9.88	0

Non-canonical half-site	RRRCWWGYYY	ΔN-P63α (RLU x 10^5)	TA-P63α (RLU x 10^5)	ΔN-P63α /TA-P63α	Total No. mismatches
CON D 1/2	GGG**CATG**CCC	0.75	ND	NA	0
CON E 1/2	GAG**CATG**TCC	ND	ND	NA	0
CON G 1/2	GGG**CATG**TCT	0.13	ND	NA	0
CON H 1/2	GGG**CTTG**TCC	ND	ND	NA	0
CON I 1/2	GGG**CAAG**TCC	ND	ND	NA	0
miR-34a RE2	GAG**CATG**CCC	0.40	ND	NA	0
miR-34a RE3	AGA**CTTG**CCT	ND	ND	NA	0
**Multimers or altered-structure**		**ΔN-P63α (RLU x 10^5)**	**TA-P63α (RLU x 10^5)**	**ΔN-P63α /TA-P63α**	**Total No. mismatches**
PG13	(AGG**CAAG**TCCAGG**CAGG**CC)$_{13}$	140.94	875.51	0.16	1 and lack of 1 base
MDM2P2C	GGT**CAAG**TTG-GGA**CACG**TCC	211.20	416.82	0.51	6
	GAG**CTAA**GTC-c-TGA**CATG**TCT				
BAX A+B	TCA**CAAG**TTA-g-AGA**CAAG**CCT	207.42	220.84	0.94	6
	AGA**CAAG**CCT-GGG**CGTG**GGC				
2XRGC	(GGA**CTTG**CCT-GGC**CTTG**CCT)$_2$	94.11	12.02	7.83	2
BAI	TGG**CTG**CCT-GGA**CATG**TTC	45.35	0.50	89.86	1 and lack of 1 base
14-3-3σ	TAG**CATTAG**CCC-AGA**CATG**TCC	48.90	ND	>89.96	1 and presence of two extra bases
PRODH +2.8	TTA**CAAG**CCC-tag-GCT**CATG**CCT-AGG**CATG**GTG-GCT**CATG**CCT-gta-ATT**CTAG**CAC	20.15	ND	>89.96	11
PRODH -0.9	CAC**CAGG**CTC-cactat-GGG**CTTG**TCT-tcgtg-TGA**CTTC**TGT	0.17	ND	NA	6

Table S2. Relative transactivation capacity and specificity of ΔN-P63β and TA-P63β towards a subset of REs tested using the yeast-based transactivation assay.

Summary of ΔN-P63β and TA-P63β transactivation capacities towards 35 REs measured using the yeast functional assay and as described in Table S1. The columns of the table report: 1) the name of the RE; 2) the transactivation ability of ΔN-P63β; 3) the transactivation ability of TA-P63β; 4) the ΔN-P63β/TA-P63β transactivation ratio. All values are expressed as RLU x 10^5. The activity of ΔN-P63β on half-site REs was not tested (Not measured). RE sequences can be found in Table S1.

**Canonical without spacer**	**ΔN-P63β (RLU x 10^5)**	**TA-P63β (RLU x 10^5)**	**ΔN-P63β /TA-P63β**
**C2 SP0**	16.54	807.40	0.02
**CON A**	9.87	730.55	0.01
**R2**	16.47	689.55	0.02
**P21-5'**	16.15	681.67	0.02
**PUMA**	14.57	661.83	0.02
**C1 SP0**	17.46	393.24	0.04
**miR-34a RE1**	21.33	552.61	0.04
**miR-198 SNP**	12.06	354.46	0.03
**miR-202**	11.22	291.13	0.04
**AIP1**	12.75	450.09	0.03
**GADD45**	15.22	268.96	0.06
**PRODH +6.8**	20.21	263.75	0.08
**miR-221**	12.25	170.06	0.07
**PCNA**	17.99	190.86	0.09
**XPC**	19.82	64.48	0.31
**PRODH +1.7**	13.85	39.63	0.35
**Canonical with spacer**	**ΔN-P63β (RLU x 10^5)**	**TA-P63β (RLU x 10^5)**	**ΔN-P63β /TA-P63β**
**C2 SP1**	9.76	284.32	0.03
**C2 SP2**	14.93	125.24	0.12
**C2 SP4**	6.85	218.33	0.03
**C1 SP4**	13.98	112.07	0.12
**PRODH +4.7**	14.37	9.14	1.57
**PRODH -3.1**	1.35	0.39	3.49
**C1 SP2**	2.32	0.63	3.70
**Non-canonical three-quarter site**	**ΔN-P63β (RLU x 10^5)**	**TA-P63β (RLU x 10^5)**	**ΔN-P63β /TA-P63β**
**miR-10b**	23.06	404.19	0.06
**CON J**	18.49	249.22	0.07
**P21-3' Complete**	17.72	294.53	0.06
**CON K**	9.07	134.90	0.07
**Non-canonical half-site**	**ΔN-P63β (RLU x 10^5)**	**TA-P63β (RLU x 10^5)**	**ΔN-P63β /TA-P63β**

**CON D 1/2**	Not measured	0.98	NA
**CON E 1/2**	Not measured	ND	NA
**CON G 1/2**	Not measured	0.11	NA
**CON H 1/2**	Not measured	ND	NA
**CON I 1/2**	Not measured	1.93	NA
**Multimers or altered-structure**	**ΔN-P63β (RLU x 10^5)**	**TA-P63β (RLU x 10^5)**	**ΔN-P63β /TA-P63β**
**BAX A+B**	21.14	836.06	0.03
**PRODH +2.8**	8.85	16.10	0.55
**PRODH -0.9**	1.14	0.34	3.32

Table S3. Relative transactivation capacity of ΔN-P73α, TA-P73α, ΔN-P73β and TA-P73β towards 14 REs tested using the yeast-based transactivation assay.

Measurements were conducted as described in Table S1. The various columns of the table report: 1) the name of the RE; 2) the transactivation ability of ΔN-P73α; 3) the transactivation ability of TA-P73α; 4) the transactivation ability of ΔN-P73β; 5) the transactivation ability of TA-P73β. All values are expressed as RLU x 10^5. While the transactivation activity of ΔN-P73α was undetectable (ND, not detectable) only on the AIP1 and XPC REs, ΔN-P73β was inactive on all REs with the exception of P21-5' towards which it showed very low activity. RE sequences can be found in Table S1.

**Canonical without spacer**	**ΔN-P73α (RLU x 10^5)**	**TA-P73α (RLU x 10^5)**	**ΔN-P73β (RLU x 10^5)**	**TA-P73β (RLU x 10^5)**
**CON A**	0.76	461.47	ND	534.75
**P21-5'**	9.63	231.50	0.67	458.97
**PUMA**	3.24	152.16	ND	347.02
**miR-34a RE1**	3.06	99.79	ND	187.13
**miR-198 SNP**	1.65	37.83	ND	117.02
**miR-202**	0.85	25.30	ND	76.62
**AIP1**	ND	63.74	ND	120.55
**GADD45**	1.43	12.23	ND	59.07
**PRODH +6.8**	1.21	16.67	ND	48.37
**miR-198**	0.04	4.04	ND	10.73
**PCNA**	0.31	4.61	ND	12.91
**XPC**	ND	0.72	ND	4.18
**PRODH +1.7**	0.37	0.99	ND	0.94
**Multimers or altered-structure**	**ΔN-P73α (RLU x 10^5)**	**TA-P73α (RLU x 10^5)**	**ΔN-P73β (RLU x 10^5)**	**TA-P73β (RLU x 10^5)**
**BAX A+B**	2.70	157.93	ND	335.77

Table S4. Relative P53 transactivation capacity towards a subset of REs.

Summary of P53 transactivation capacity towards the 35 REs tested in Table S1 and S2. Measurements were conducted as described in Table S1. The columns of the table report: 1) the name of the RE; 2) the transactivation ability (RLU x 10^5) of P53. RE sequences can be found in Table S1.

**Canonical site without spacer**	**P53 (RLU x 10^5)**
**C2 SP0**	1239.86
**CON A**	1218.95
**R2**	1031.75
**P21-5'**	1130.08
**PUMA**	996.64
**C1 SP0**	903.85
**miR-34a RE1**	1186.63
**miR-198 SNP**	593.19
**miR-202**	828.29
**AIP1**	828.89
**GADD45**	676.77
**PRODH +6.8**	413.26
**miR-221**	215.15
**PCNA**	684.91
**XPC**	126.63
**PRODH +1.7**	188.90
**Canonical with spacer**	**P53 (RLU x 10^5)**
**C2 SP1**	1400.82
**C2 SP2**	865.20
**C2 SP4**	1003.52
**C1 SP4**	198.55
**PRODH +4.7**	307.20
**PRODH -3.1**	0.89
**C1 SP2**	195.84
**Non-canonical three-quarter site**	**P53 (RLU x 10^5)**
**miR-10b**	463.86
**CON J**	637.12
**P21-3' Complete**	1012.81
**CON K**	563.18
**Non-canonical half-site**	**P53 (RLU x 10^5)**
**CON D 1/2**	235.25
**CONE 1/2**	12.12
**CON G 1/2**	134.33
**CON H 1/2**	29.69
**CON I 1/2**	12.93
**Multimers or altered-structure**	**P53 (RLU x 10^5)**
**BAX A+B**	1352.23
**PRODH +2.8**	232.31
**PRODH -0.9**	1.25

Table S5. Relative transactivation values of 5 REs that exhibited comparable or even higher responsiveness to ΔN-P63α, TA-P63β than to P53.

Measurements were conducted as described in Table S1. The columns of the table report: 1) the name of the RE; 2) the transactivation ability of ΔN-P63α; 3) the transactivation ability of TA-P63β; 4) the transactivation ability of P53. All values are expressed as RLU x 10^5. RE sequences can be found in Table S1.

**Canonical site without spacer**	**ΔN-P63α (RLU x 10^5)**	**TA-P63β (RLU x 10^5)**	**P53 (RLU x 10^5)**
**P48**	15.12	36.69	9.15
**PERP**	105.40	137.00	33.60
**COL18A1 ISOFORM2**	56.72	64.85	49.35
**H2**	43.15	42.01	39.87
**miR-198**	78.54	137.82	18.82
